# Interaction Between Plant Competition and Rhizospheric Bacterial Community Influence Secondary Succession of Abandoned Farmland on the Loess Plateau of China

**DOI:** 10.3389/fpls.2018.00898

**Published:** 2018-07-12

**Authors:** Caili Sun, Guobin Liu, Sha Xue

**Affiliations:** ^1^State Key Laboratory of Soil Erosion and Dryland Farming on the Loess Plateau, Northwest A&F University, Yangling, China; ^2^College of Eco-Environmental Engineering, Guizhou Minzu University, Guiyang, China; ^3^Institute of Soil and Water Conservation, Chinese Academy of Sciences and Ministry of Water Resources, Yangling, China

**Keywords:** 16S rRNA sequencing, grass, pot experiment, plant competition, plant-soil interaction

## Abstract

Interactions between plant and soil communities have important implication for plant competition, development and succession. In order to explore the internal mechanism behind natural succession of abandoned farmland, we test the effect of plant–soil interaction on plant growth and competitive ability through performing a pot experiment, which included three grasses in different successional stages on the Loess Plateau of China (*Setaria viridis, Stipa bungeana*, and *Bothriochloa ischaemum*) in monoculture and all possible two- and three-way combinations, along with a plant-free control pot. The plants were harvested after about 4 months, and the rhizospheric soil was collected. The bacterial communities of the soils were analyzed by high-throughput sequencing of the 16S rRNA gene. Plant competition affected richness of bacterial communities. *Proteobacteria* and *Bacteroidetes* were generally higher and *Actinobacteria* and *Acidobacteria* were lower in relative abundance in the mixed treatments associated with *B. ischaemum*. Photosynthetic bacterium, Genus *Rhodobacter* family *Rhodospirillaceae*, affected the growth condition and increased the competitive ability of *B. ischaemum*. Differences in the amounts of soil organic carbon, water-soluble organic carbon and nitrate nitrogen and available phosphorus drove the differences in bacterial communities. Our study has an important significance for understanding the trend of natural succession on the abandoned farmland on the Loess Plateau of China.

## Introduction

Plants supply litter, decomposing roots and exudates that provide distinct combinations of substrates to unique soil microbial and macro-invertebrate communities, and the soil community in turn influence nutrient cycling, plant nutrient availability, disease protection, plant health, and plant growth (Andrews et al., 2010; Aguilera et al., 2011; McHugh and Schwartz, 2015). The interactions between plant and soil communities have an effect on plant abundance and competitive ability (Kuramae et al., 2011). Further, plant competition has been reported to be fundamental for determining plant community composition and successional dynamics (Goldberg and Barton, 1992; Aguilera et al., 2017). Kardol et al. (2006) observed negative, neutral, and positive plant–soil feedbacks for early-, mid-, and late-successional plant species, respectively, and suggested that negative feedbacks among early successional species accelerate succession while positive feedbacks among late-successional species stabilize communities. The abiotic and biotic factors within plant–soil system driving vegetation succession has been widely studied (Reynolds et al., 2003; Kulmatiski et al., 2008; Kuramae et al., 2011), however, it has remained unknown whether changes in soil communities enhance or retard vegetation changes, and whether microbial factors contribute to plant growth and competition in a shorter term.

The most pronounced effects of plant on soil microbial community was observed in the zone directly adjacent to active plant roots, i.e., the rhizosphere (Berg and Smalla, 2009). The rhizosphere is a critical interface supporting the exchange of resources between plants and their associated soil environments and is home to an overwhelming number of microorganisms and invertebrates (Peiffer et al., 2013; Philippot et al., 2013). Microbial communities in the rhizosphere perform fundamental processes that greatly affect nutrient cycling, plant growth and root health (Marschner et al., 2004; Philippot et al., 2013) and potentially represent a mechanistic link between plant diversity and ecosystemic function (Zak et al., 2003; Singh et al., 2004). Understanding the relationships between plant species and rhizospheric bacterial communities is thus important for predicting the response of plant growth and competition to rhizospheric microbial community.

Plant succession is often associated with plant competition, changes of plant species, plant diversity, and plant community composition, and have been shown to influence rhizospheric microbial communities (Felske et al., 2000; Kardol et al., 2006; Kuramae et al., 2011). van de Voorde et al. (2011) indicated that plant–soil interactions may enhance the rate of succession through priority effects, and intra- and interspecific plant–soil interactions can prioritize transitions of plant species in plant communities. On the Loess Plateau of China, Zhang et al. (2016) suggested that both below- and aboveground underwent secondary succession after farmland was abandoned, but below- and aboveground communities had incongruous process during the succession. However, study of Zhang et al. (2016) just found the incongruous successional process of below- and aboveground communities, reasons and internal mechanism driving this phenomenon has not been clearly and deeply illustrated.

Given all of this, we simulated changes in species community across different successional stages using three grass species through pot experiment and hypothesized that plant competition and rhizospheric bacterial community influenced each other during succession. Three grass species, *Setaria viridis, Stipa bungeana*, and *Bothriochloa ischaemum*, were selected, which are typical native herbaceous species widely distributed on the Loess Plateau of China (Ning et al., 2008; Liu and Wang, 2009). *S. viridis* is dominant at the early successional stage but is replaced by *S. bungeana* and *B. ischaemum* in the middle and later stages (Zhang, 2005; Wang, 2006; Wang et al., 2009). With the help of high-throughput sequencing method, we aimed to (1) analyze the effect of dominant species and plant competition on soil community; (2) explore the role of bacterial communities played in the plant growth, competition and succession. Our study will have important significance for understanding the internal mechanism behind vegetation succession of abandoned farmland on the Loess Plateau of China.

## Materials and Methods

### Pot Experiment

#### Experimental Site

An outdoor controlled experiment was conducted at the Institute of Soil and Water Conservation, Chinese Academy of Sciences, in Yangling, Shaanxi Province, China (34°12′N, 108°07′E, 530 m a.s.l.). This area has a warm continental monsoon climate with hot and wet summers and cold and dry winters. The mean annual temperature is 13.0°C, with maximum and minimum mean monthly temperatures of 26.7°C in July and -1 to -2°C in January. The mean annual precipitation is about 632 mm, which occurs mainly from July to September (Xu et al., 2011).

#### Plant Material

Three gramineous plants, *S. viridis, S. bungeana*, and *B. ischaemum*, were selected for this study. All are typical native herbaceous grasses widely distributed on the Loess Plateau of China (Ning et al., 2008; Wang et al., 2009; Wang et al., 2011). *S. viridis* is an annual grass, and *S. bungeana* and *B. ischaemum* are perennial grasses. *S. viridis* and *S. bungeana* are C3 plants, and *B. ischaemum* is a C4 plant. Seeds were collected in autumn 2014 from a field experimental station (Ansai Research Station) of the Institute of Soil and Water Conservation on the plateau.

#### Experimental Design

We used loessial soil (Calcic Cambisol) collected from farmland at the Ansai Research Station. The field capacity of the loess, determined as gravimetric soil-moisture content, was about 20%. The soil at the beginning of the experiment had a pH of 8.68 and contained 1.71g kg^-1^ soil organic carbon (SOC), 0.22 g kg^-1^ total nitrogen (TN), 0.55g kg^-1^ total phosphorous (TP) and 3.04 mg kg^-1^ available phosphorous (AP). The soil was added to cylindrical polyvinyl chloride (PVC) pots (height: 20 cm; diameter: 15 cm). The bottom of each pot was covered with about 1 kg of stones (diameter: 1 cm). A plastic tube (diameter: 1 cm; length 25 cm) used for irrigation was placed vertically immediately above the stones. Filter paper was then placed over the stones and around the plastic tube. The pot was then filled with about 3.8 kg of sieved (5 mm) and air-dried loess.

**FIGURE 1:**
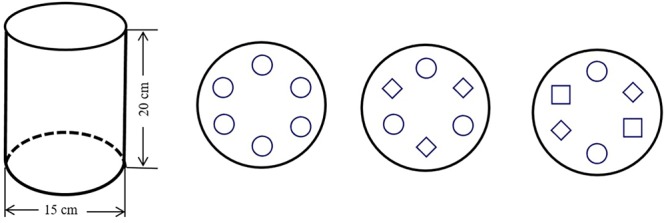
Size of experimental pot and schematic diagram of the experimental design. Symbols ○, ♢, and □ represent two *Setaria viridis*, one *Stipa bungeana*, and one *Bothriochloa ischaemum*, respectively.

We tested eight treatments: *S. viridis, S. bungeana*, and *B. ischaemum* as monocultures (G, C, and B, respectively), all pair-wise combinations of these three species (GC, CB, and GB), all species mixed (GCB) and pots without plants as a control (CK). Each treatment had five replicates, for a total of 40 pots. The planted pots were sown with multiple seeds on 22 April 2015, in order to simulate the field condition, the plants were thinned to six *S. bungeana* and *B. ischaemum* and twelve *S. viridis* in the monocultures; three *S. bungeana*, three *B. ischaemum* or six *S. viridis* in the pair-wise combinations and two *S. bungeana* and *B. ischaemum* and four *S. viridis* in GCB to simulate natural proportions. The pot size and planting pattern were shown in **Figure 1**. We watered all pots with distilled water to maintain near 80% field capacity by weighting method until the end of the experiment, in order to minimize the effect of moisture on plant and microbial community. The plants grew normally in outdoor conditions under a rain shelter (height 3 m) to prevent the addition of rainwater. Pot positions were changed once a week to reduce any micro-environmental effects.

### Sample Collection and Analysis

#### Sample Collection

Three of the five replicates were randomly selected for sampling 117 days after sowing. Above- and belowground tissues were harvested separately. The roots of the competing plant species tended to become entangled, so we collected all roots together. Loosely adhering soil was removed by shaking and was discarded, and the remaining soil strongly adhering to the roots was considered the rhizosphere and was brushed from the entire root system for collection. The above- and belowground tissues were carefully washed with distilled water and were then oven-dried at 80°C for 48 h for determining their biomass dry weights. Visible plant roots and debris were removed from each soil sample, which was then divided into three parts. One part was immediately stored at -80°C for DNA analysis, the second part was immediately sieved through a 2-mm mesh and stored at 4°C for the analysis of water-soluble organic carbon (WSOC) and the third part was air-dried for physicochemical analysis.

#### Soil Chemical Parameters

Soil organic carbon content was determined by the Walkley–Black method (Nelson and Sommers, 1982). WSOC content was determined following the method described by Sims and Haby (1971). Briefly, 120 ml of distilled water were added to 40 g of fresh soil, the aqueous solution (1:3 soil:water) was mechanically shaken for 1 h, followed by centrifugation at 5,000 rpm for 15 min and filtration through a 0.45-μm membrane. The WSOC content was then determined using a Liqui TOCII analyzer (Elementar, Germany). TN content was determined using the Kjeldahl method (Bremner and Mulvaney, 1982). Ammonium nitrogen (NH_4_^+^-N) and nitrate nitrogen (NO_3_^-^-N) were extracted from 2.5 g of soil incubated in 25 ml 1 M KCl for 1 h and analyzed calorimetrically on an Alpkem Autoanalyzer (OI Analytical, College Station, TX, United States). TP content was determined by melt-molybdenum, antimony and scandium colorimetry (Murphy and Riley, 1962), and AP content was determined by the Olsen method (Olsen and Sommers, 1982). Soil pH was determined by an automatic titrator (Metrahm 702, Switzerland) in 1:2.5 soil:water suspensions.

#### DNA Extraction and Sequencing

##### DNA extraction and illumina HiSeq high-throughput sequencing

DNA was extracted from 0.25 g of soil using a TIANamp Soil DNA Kit [Tiangen Biotech (Beijing) Co., Ltd, Beijing, China]. The DNA extracts were diluted 10-fold and spectrophotometrically assessed for quality and quantity (NanoDrop ND-1000, NanoDrop Technologies, Wilmington, DE, United States). The integrity of the DNA extracts was confirmed by 1% agarose gel electrophoresis. DNA was amplified by PCR in triplicate using primers for the 16S rRNA gene. The 341F (5′ - CCTAYGGGRBGCASCAG - 3′) and 806R (5′ - GGACTACNNGGGTATCTAAT - 3′) primers were designed to amplify the hypervariable V3+V4 region of bacterial 16S rRNA genes. The primers were tagged with unique barcodes for each sample. PCR reactions were performed in a volume of 30 μl containing 2 μl of sterile ultrapure water, 15 μl of Phusion Master Mix (2X), 3 μl of 6 μM primers and 10 μl of template DNA (5–10 ng). The PCR program was: 98°C for 60 s; 30 cycles of 98°C for 10 s, 50°C for 30 s and 72°C for 30 s; 72°C for 5 min and hold at 4°C. Sterile ultrapure water was used as negative controls for detecting primer or DNA contamination. Successful PCR amplification was verified by 2% agarose gel electrophoresis. The triplicate PCR products were mixed in equidensity ratios and were then purified using a Qiagen Gel Extraction Kit (Qiagen, Germany). Sequencing libraries were generated using a TruSeq^®^ DNA PCR-Free Sample Preparation Kit (Illumina, United States) following the manufacturer’s recommendations, and index codes were added. The quality of the libraries was assessed using a Qubit@ 2.0 Fluorometer (Thermo Scientific) and an Agilent Bioanalyzer 2100 system. The libraries were then sequenced on an Illumina HiSeq 2500 platform, generating 250-bp paired-end reads.

##### Processing of sequencing data

The sequences were quality-filtered and chimera checked using the Quantitative Insights Into Microbial Ecology (QIIME) workflow (Caporaso et al., 2010). In brief, quality filtering of the raw tags was performed under specific conditions to obtain high-quality clean tags (Bokulich et al., 2013) following QIIME quality control (Caporaso et al., 2010). Sequences with the same barcode were sorted into the same sample (Edgar et al., 2011). The tags were compared with a reference database (Gold database^1^ using the UCHIME algorithm (Caporaso et al., 2010) to detect chimeric sequences, which were then removed to obtain the effective tags. The remaining sequences were clustered by Uparse (Edgar, 2013) and assigned to operational taxonomic units (OTUs) at similarities of 97%. The Chao1 estimator and the abundance-based coverage estimator (ACE) were calculated as indices of community diversity, and rarefaction curves were obtained using QIIME (Version 1.7.0) and displayed with R (R Development Core Team, 2004). Taxonomic information for each representative sequence was obtained from the GreenGene Database (DeSantis et al., 2006) using the RDP classifier (Wang et al., 2007) algorithm.

### Data Analysis

#### Indices of Plant Competition

The relative yield total (*RYT*) was defined and developed by De Wit (1960) and Snaydon (1991), and was used almost universally to measure the extent to which components of a mixture compete for common limiting resources. *RYT* values are calculated by the following expression:

(1) $$\begin{array}{c} RYT=\frac{Y_{ij}}{Y_{ii}}+\frac{Y_{ji}}{Y_{jj}} \end{array}$$

where *Y_ii_* and *Y_jj_* are the aboveground biomass of components *i* and *j* when grown in monoculture, and *Y_ij_* and *Y_ji_*, are the aboveground biomass of the components when grown in mixtures with each other. *RYT* = 1 indicates species compete for the same resources and show no resource complementarity; *RYT* > 1 indicates species compete partially and show partial resource complementarity; *RYT* < 1 indicates there is mutual antagonism.

The competitive ratio (*CR*) was propose by Willey and Rao (1980), and used to evaluate the exact degree of competition, which was calculated as:

(2) $$\begin{array}{c} CR_{ij}=(\frac{Y_{ij}}{Y_{ii}}÷\frac{Y_{ji}}{Y_{jj}})\times \frac{P_{j}}{P_{i}} \end{array}$$

where, means of *Y_ii_, Y_jj_, Y_ij_*, and *Y_ji_* are the same as formula (1). *P_i_* and *P_j_* are the proportion of *i* and *j* was sown, respectively. *CR_ij_*> 1 means the competitive ability of *i* is greater than that of *j*; *CR_ij_* < 1 means *i* is less competitive than *j*.

#### Statistical Analysis

Differences in the soil chemical properties and microbial diversities among treatments were assessed by a one-way analysis of variance and multifractal comparison, and the means were compared by Duncan’s tests at *P* < 0.05. The Shannon and Simpson indices were calculated to assess microbial diversity, and Chao1 and ACE were calculated to estimate species richness. Principal coordinates analysis (PCoA) was used to evaluate the overall differences in the structures of the microbial communities based on Unweighted Unifrac distances. A canonical correspondence analysis (CCA) with forward selection and 999 Monte Carlo simulations was used to identify soil chemical properties that significantly explained the variance of the microbial groups (*P* < 0.05). Structural equation modeling (SEM) was used to gain a mechanistic understanding of the effect of the plant and soil properties on rhizospheric bacterial composition and richness. The structures of the bacterial communities were assessed by the first principal components of the PCoA. We chose to use root biomass as the original causal factor, because the effects of roots on a rhizosphere, such as exudates and root death, are likely the most important influences on the rhizospheric chemical properties and microbial communities (Lamb et al., 2010; Philippot et al., 2013). The data in the SEM analysis were fitted to the models using the generalized least squares method. Adequate model fits were indicated by the χ^2^ test (*df* > 5; *P* > 0.05) and a low root mean squared error of approximation (Grace, 2006). Statistical tests were performed using the *vegan* and *sem* packages in R v.3.3.0 (R Development Core Team, 2004).

## Results

### Plant Competitive Characteristic and Its Effect on Plant Biomass and Soil Chemical Properties

The species had different biomasses, and increasing the number of species had only occasional additive effects on biomass. Aboveground and total biomasses were the highest in the B treatment, but belowground biomass followed the order B > CB, GB and GCB > G, C and GC (**Table 1**). According to formulas (1) and (2), the *RYT* in mixture treatments *Setaria viridis + Stipa bungeana, Stipa bungeana + Bothriochloa ischaemum*, and *Stipa bungeana + Bothriochloa ischaemum* were greater than 1, but the competitive ratios (CRs) in mixture treatments were less than 1 (**Table 2**).

**Table 1:** Mean plant dry weights (g) in the treatments.

Treatment	Aboveground	Belowground	Total
	dry weight	dry weight	dry weight
CK	–	–	–
G	1.211ab	0.289c	1.500cd
C	0.758c	0.240c	0.998d
B	1.500a	1.271a	2.771a
GC	1.004bc	0.367c	1.371cd
CB	1.207ab	1.133ab	2.340ab
GB	1.347ab	0.949ab	2.297ab
GCB	1.127abc	0.826b	1.953bc

*Different letters within a column indicate significant differences at *P* < 0.05. CK, control; G, *S. viridis* monoculture; C, *S. bungeana* monoculture; B, *B. ischaemum* monoculture; GC, *S. viridis* + *S. bungeana*; CB, *S. bungeana* + *B. ischaemum*; GB, *S. viridis* + *B. ischaemum*; GCB, *S. viridis* + *S. bungeana* + *B. ischaemum*.*

**Table 2:** The competitive intensity of three grass species.

Pattern of mixture	*Y_ij_*	*Y_ii_*	*Y_ji_*	*Y_jj_*	*RYT*	*CR_ij_*
GC (*Setaria viridis + Stipa bungeana*)	0.127 ± 0.040	0.101 ± 0.019	0.080 ± 0.042	0.110 ± 0.017	1.992 ± 0.081	0.865 ± 0.251
CB (*Stipa bungeana + Bothriochloa ischaemum*)	0.051 ± 0.007	0.110 ± 0.017	0.352 ± 0.065	0.250 ± 0.015	1.869 ± 0.271	0.328 ± 0.145
GB (*Setaria viridis + Bothriochloa ischaemum*)	0.076 ± 0.005	0.101 ± 0.019	0.297 ± 0.045	0.250 ± 0.015	1.942 ± 0.134	0.317 ± 0.140

*Y_*ii*_ means aboveground biomass of species *i* in monoculture; *Y_*jj*_* means aboveground biomass of species *j* in monoculture; *Y_*ij*_* means aboveground biomass of species *i* in presence of species *j*; *Y_*ji*_* means aboveground biomass of species *j* in presence of species *i*; *RYT* means the relative yield total; *CR_*ij*_* means the competitive ratio.*

Soil organic carbon and WSOC contents were higher in the planted treatments than CK, but the mixed treatments almost showed non-additive effect compared to the monocultured treatments. Trends were opposite between AP and NO_3_^-^-N content and SOC and WSOC contents. TN contents and pH were occasionally higher in the planted treatments relative to CK (**Table 3**).

**Table 3:** Mean soil chemical properties in the treatments.

Treatment	SOC (g kg^-1^)	WSOC (mg kg^-1^)	TN (g kg^-1^)	NH_4_^+^-N (mg kg^-1^)	NO_3_^-^-N (mg kg^-1^)	TP (g kg^-1^)	AP (mg kg^-1^)	pH
CK	1.744c	61.182	0.225b	7.212	4.320a	0.557ab	3.320a	8.683cd
G	1.792bc	66.946	0.232ab	7.457	2.773c	0.556ab	1.648c	8.748ab
C	1.793bc	65.523	0.235ab	7.197	3.028bc	0.535b	1.862bc	8.780a
B	1.834abc	69.707	0.225b	8.030	3.237bc	0.567a	1.699c	8.660d
GC	1.850ab	69.016	0.225b	7.910	3.330bc	0.562a	1.971bc	8.735ab
CB	1.867ab	71.783	0.235ab	7.558	3.470bc	0.552ab	2.269b	8.720bc
GB	1.897a	67.642	0.244a	7.315	2.777c	0.555ab	2.169b	8.732abc
GCB	1.882ab	68.100	0.229b	7.077	3.747ab	0.544ab	2.305b	8.715bc

*Different letters within a column indicate significant differences at *P* < 0.05. CK, control; G, *S. viridis* monoculture; C, *S. bungeana* monoculture; B, *B. ischaemum* monoculture; GC, *S. viridis* + *S. bungeana*; CB, *S. bungeana* + *B. ischaemum*; GB, *S. viridis* + *B. ischaemum*; GCB, *S. viridis* + *S. bungeana* + *B. ischaemum*.*

### Diversity and Structure of Microbial Communities in the Treatments

#### Bacterial α-Diversity

**FIGURE 2:**
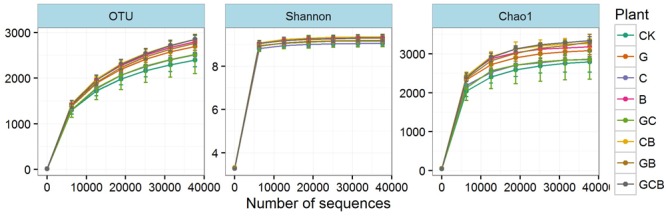
Rarefaction curves for operational taxonomic units (OTUs) and the Shannon and Chao1 indices in the treatments. Error bars indicate means ± standard error (*n* = 3).

High-throughput sequencing produced a total of 1,436,760 high-quality sequences and a total of 64,218 OTUs with 1,934-3,001 OTUs per sample after removing low-quality reads and chimeras and trimming the primers and barcodes. The Good’s coverage values had a range of 0.981–0.986 at 97% similarity cutoff (**Table 4**). The rarefaction curves for the OTUs, Shannon index and Chao1 reached an asymptote (**Figure 2**), indicating that current numbers of sequence reads were sufficient to capture the bacterial diversity in these soils.

**Table 4:** Mean α-diversities of the microbial communities in the treatments.

Treatment	Richness		Diversity		OTUs	Coverage (%)
	Chao1	ACE	Shannon	Simpson		
CK	2791^b^	2843^b^	9.173	0.995^ab^	2399^b^	0.986
G	3084^ab^	3160^ab^	9.308	0.995^a^	2698^ab^	0.983
C	2861^ab^	2923^ab^	9.058	0.994^ab^	2516^ab^	0.985
B	3179^ab^	3270^ab^	9.332	0.995^ab^	2799^ab^	0.983
GC	2856^ab^	2937^ab^	9.183	0.995^ab^	2520^ab^	0.985
CB	3266^ab^	3367^ab^	9.364	0.994^ab^	2853^a^	0.982
GB	3305^ab^	3334^ab^	9.205	0.993^b^	2770^ab^	0.981
GCB	3344^a^	3433^a^	9.311	0.994^ab^	2851^a^	0.981

*Different letters within a column indicate significant differences at *P* < 0.05. ACE, abundance-based coverage estimator; OTUs, operational taxonomic units; CK, control; G, *S. viridis* monoculture; C, *S. bungeana* monoculture; B, *B. ischaemum* monoculture; GC, *S. viridis* + *S. bungeana*; CB, *S. bungeana* + *B. ischaemum*; GB, *S. viridis* + *B. ischaemum*; GCB, *S. viridis* + *S. bungeana* + *B. ischaemum*.*

Microbial richness (Chao1 and ACE) and number of OTUs were significantly higher only in GCB treatment compared to CK. Neither plant species nor plant competition significantly affected microbial diversity (Shannon and Simpson indices) (**Table 5**).

**Table 5:** Results of the canonical correspondence analysis (CCA) variance partitioning of environmental factors to microbial communities.

Forward selection of variables	CCA1	CCA2	*R*^2^	*P*
SOC content	0.994	0.114	0.527	0.002**
WSOC content	0.306	–0.952	0.276	0.033*
Total N content	0.996	0.089	0.065	0.498
NH_4_^+^-N content	–0. 935	–0.354	0.007	0.926
NO_3_^-^-N content	–0.470	0.883	0.281	0.036*
TP content	–0.981	–0.192	0.011	0.870
AP content	–0.117	0.993	0.395	0.004**
pH value	–0.858	–0.514	0.096	0.322

*^∗^*P* < 0.05; ^∗∗^*P* < 0.01.*

#### Bacterial-Community Composition and Structure

**FIGURE 3:**
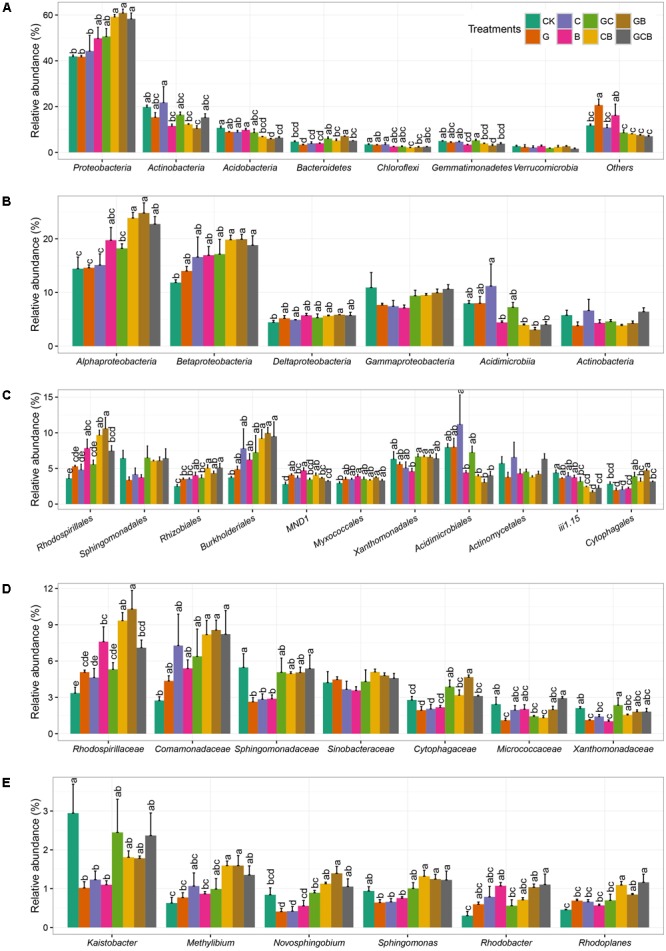
Relative abundance of dominant rhizospheric bacterial community at the phylum level **(A)**, class level **(B)**, order level **(C)**, family level **(D)**, and genus level **(E)** within different treatments. Different letters within a class indicate significant differences at *P* < 0.05. No letters indicate no significant difference within a phylum. Error bars indicate the standard error of relative abundance between three replicate samples. CK, control; G, *S. viridis* monoculture; C, *S. bungeana* monoculture; B, *B. ischaemum* monoculture; GC, *S. viridis* + *S. bungeana*; CB, *S. bungeana* + *B. ischaemum*; GB, *S. viridis* + *B. ischaemum*; GCB, *S. viridis* + *S. bungeana* + *B. ischaemum*.

The rhizospheric bacterial taxa differed among various competi-tive treatments (**Figure 3**). The dominant phyla were *Proteobacteria* (average 50.82%), *Actinobacteria* (15.36%), *Acidobacteria* (8.29%), *Bacteroidetes* (4.91%), *Gemmatimona-detes* (4.15%), *Verrucomicrobia* (2.29%), and *Chloroflexi* (2.78%). Effects of plant competition on bacterial composition was stronger than plant species. The relative abundance of *Proteobacteria* were significantly higher in CB, GB, and GCB treatments than in G, C, and CK treatments, and relative abundance of *Bacteroidetes* was much higher in mixed treatments than in monocultured and CK treatments. By contrast, the relative abundance of *Actinobacteria* in B, CB, and GB was significantly lower than CK treatment, and the relative abundance of *Acidobacteria* was lower in the mixed than in the monocultured and CK treatments. The abundances of the other phyla just differed occasionally among treatments (**Figure 3A**).

The classes *Alpha-, Beta-, Delta-*, and *Gammaproteobacteria*, all part of the most dominant phylum *Proteobacteria*, accounted for an average of 19.2, 16.89, 5.35, and 9.09% of the total populations, respectively, moreover, *Acidimicrobiia* and *Actinobacteria* had average relative abundance of 6.24 and 4.97%, respectively. *Alpha-* and *Betaproteobacteria* differed markedly in abundance among the treatments, which were higher in CB, GB, and GCB treatments compared to the other treatments. The relative abundance of *Acidimicrobiia* in B, CB, GB, and GCB treatments were lower compared to the other treatments (**Figure 3B**).

Dominant orders of *Rhodospirillales, Rhizobiales, Burkholderiales, Acidimicrobiales, iii1-15*, and *Cytophagales* showed remarkable difference among treatments. *Rhodospirillales, Rhizobiales, Burkholderiales*, and *Cytophagales* generally had higher relative abundances in CB, GB, and GCB treatments than the monocultured treatments and CK, whearas *Acidimicrobiales* and *iii1-15* showed the opposite pattern (**Figure 3C**). At the family level, *Rhodospirillaceae, Comamonadaceae*, and *Cytophagaceae* had generally higher relative abundance in CB, GB, and GCB treatments compared to monocultured and CK treatments, and relative abundance of *Sphingomonadaceae* was higher in mixed treatments than in monocultured treatments (**Figure 3D**). All of dominant genuses displayed in **Figure 3E** showed similar trends, especially *Methylibium, Novosphingobium*, and *Sphingomonas*, relative abundance of which were higher in CB, GB, and GCB treatments than in monocultured and CK treatments.

#### Bacterial β-Diversity Analysis at the OTU-Level

**FIGURE 4:**
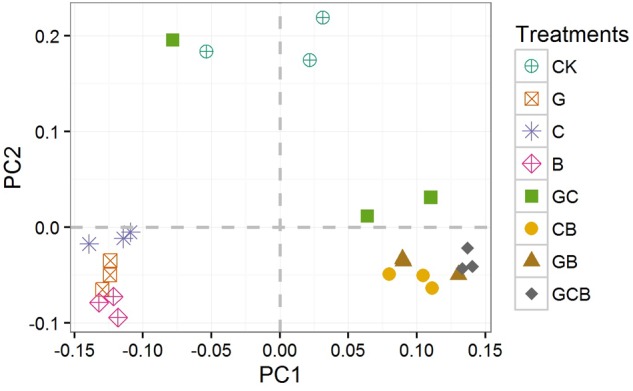
Principal coordinates analysis (PCoA) of bacterial community based on Unweighted Unifrac distances. CK, control; G, *S. viridis* monoculture; C, *S. bungeana* monoculture; B, *B. ischaemum* monoculture; GC, *S. viridis* + *S. bungeana*; CB, *S. bungeana* + *B. ischaemum*; GB, *S. viridis* + *B. ischaemum*; GCB, *S. viridis* + *S. bungeana* + *B. ischaemum*.

The PCoA clustered the bacterial communities into three distinct groups: the monocultured treatments, the mixed treatments and CK. Bacterial communities from the same planting pattern tended to group together, and showed strong clustering between samples from similar planting patterns (**Figure 4**).

### Relationships Between Soil Properties and Bacterial Communities

**FIGURE 5:**
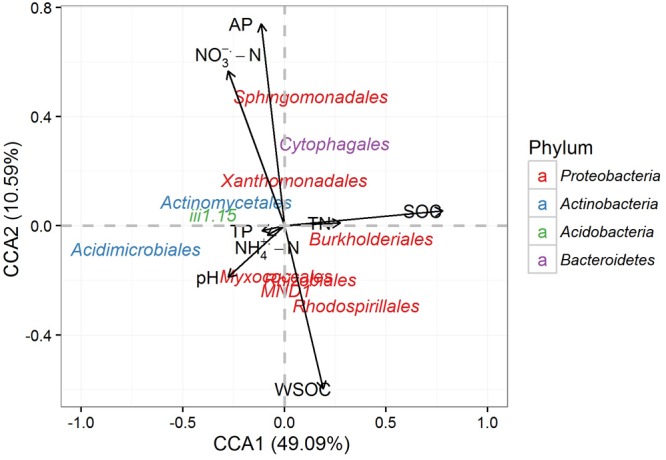
Canonical correspondence analysis (CCA) illustrating the relationships of the soil properties and the dominant rhizospheric bacterial orders.

The CCA analysis indicated that the first two canonical axes explained a total of 60.49% of the total variance, with the first and second axes explaining 49.09 and 10.59%, respectively (**Figure 5**). SOC, WSOC, NO_3_^-^-N and AP contents were the most influential factors driving the differences in community structure (**Table 3**). The vectors on the CCA ordination plot generally suggested that most subgroups of *Proteobacteria* were positively correlated with carbon contents, and the dominant subgroups of *Actinobacteria* and *Acidobacteria* were opposite. *Sphingomonadales, Xanthomonadales*, and *Cytophagales* was positively correlated with NO_3_^-^-N and AP contents.

**FIGURE 6:**
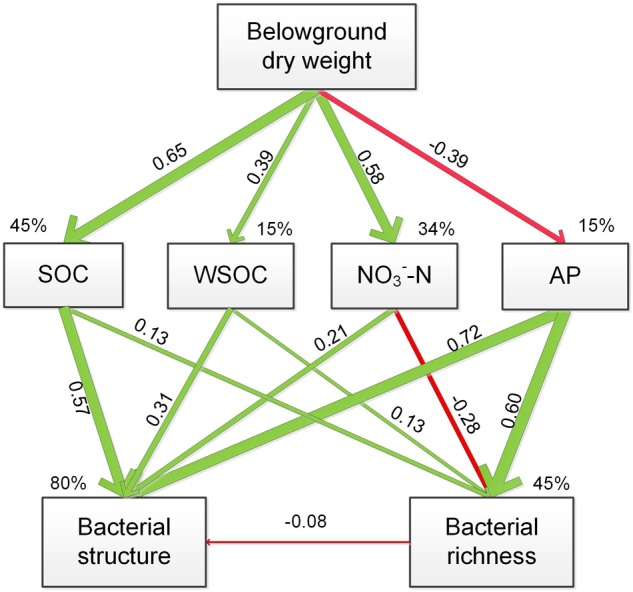
Structural equation modeling of the effect of plant composition on the plant/soil-microbe system, indicating the relationships between soil SOC, WSOC, and AP contents with bacterial structure and richness. The final model fit the data well, with χ^2^= 4.981, *df* = 8, *P* = 0.760. The red arrow indicates a significant negative correlation, and green arrows indicate positive correlations. The widths of the arrows are proportional to the path coefficients on the arrows. Percentages with the variables indicate the variance explained by the model (*R*^2^).

The soil-nutrient indices used in the SEM was selected based on the CCA analysis and identified large influences on the rhizospheric bacterial communities. The SEM model fit the data well (χ^2^= 4.981; *P* = 0.760) and accounted for 45, 15, 34, and 15% of the variability in SOC, WSOC, NO_3_^-^-N and AP contents, respectively (**Figure 6**). Belowground dry weight showed positive effect on SOC, WSOC, NO_3_^-^-N contents and negatively affected AP content. Change of bacterial structure were resulted from the direct and positive effects of SOC, WSOC, NO_3_^-^-N, and AP content, and change of bacterial richness was resulted from the positive effect of SOC, WSOC, and AP contents and negative effect of NO_3_^-^-N content.

## Discussion

### Effect of Plant Competition on Rhizospheric Bacterial Community

We saw no evidence that bacterial diversity was affected by plant species and their competition, but the richness of the bacterial community were increased in treatments of B, CB, GB even though not significant, and were significantly increased in treatment of GCB compared to CK. It has been widely accepted that plant species had an effect on microbial diversity (Ladygina and Hedlund, 2010; Burns et al., 2015). However, it is not surprising that *S. viridis, S. bungeana*, and *B. ischaemum* and their competitive treatments showed similar rhizospheric bacterial diversity, because these three grass species belong to Poaceae, and the competitive time of grass was too short to establish and generate rhizospheric bacterial community that was in contact with focal plant root system. Higher bacterial richness indices in treatments of B, CB, GB, and GCB than CK treatment was mainly due to the high inputs of readily assimilable organic substrates from the roots and the development of opportunistic populations (Marschner et al., 2004; Nielsen et al., 2011), and the higher SOC and WSOC contents in these treatments were consistent with this interpretation (**Table 3**). It is widely acknowledge that plants produce different amounts and compositions of carbon resources (Berg and Smalla, 2009; Ladygina and Hedlund, 2010), with a large proportion of the carbon in the form of water-soluble substances such as sugars, organic acids, and amino acids (Marschner et al., 2004).

As we expected, plant species and competition had an effect on rhizospheric bacterial composition in different levels. Regardless of treatments, *Proteobacteria* and *Actinobacteria* were in relatively high abundance (50.82 and 15.36%, respectively), which was in agreement with previous studies (Qiu et al., 2012; Li et al., 2015; Zhang et al., 2016), showing that they are two ubiquitous and common bacterial groups in soil. Of the four sub-groups of *Proteobacteria* phyla, *Alpha-* and *Betaproteobacteria* was more prevalent, which was in keeping with other findings (de Cárcer et al., 2007; Zhang et al., 2016). Within treatments, plant competitive treatments, especially CB, GB and GCB, generally increased the relative abundances of *Proteobacteria* and *Bacteroidetes*, and decreased the relative abundances of *Actinobacteria* and *Acidobacteria* compared to the monocultured and CK treatments. PCoA analyses confirmed that bacterial communities in the mixed treatments were different from the monocultured and CK treatments (**Figure 4**). The nutrient content was lower and nutrient availability was poorer in our study compared to soil that has been restored for many years (Zhang et al., 2016). *Proteobacteria* and *Bacteroidetes* have copiotrophic characteristics and become abundant when labile substrates are available (Fierer et al., 2007), but *Actinobacteria* and *Acidobacteria* are oligotrophic groups and prefer nutrient-poor environments (Fierer et al., 2007; McHugh and Schwartz, 2015). Thus, oligotrophs outcompete copiotrophs in competitive treatments mainly due to the presence of *B. ischaemum*, which was perennial and C4 grass species having stronger capacity for photosynthesis and releasing root exudates (Kowalchuk et al., 2002), and finally affected soil-nutrient contents in rhizosphere (Marschner et al., 2004; McHugh and Schwartz, 2015). Plant competition generally changed bacterial communities suggesting that plant combination were more effective than single species for driving the transitions of belowground bacterial communities from slow-growing oligotrophic groups to fast-growing copiotrophic groups.

Differences of *Proteobacteria* and *Acidobacteria* among treatments was mainly caused by changes of *Alpha- and Betaproteobacteria* and *Acidimicrobiia*. In order level, plant competition facilitated the increase of *Rhodospirillales, Rhizobiales, Burkholderiales*, and *Cytophagales*, and hindered the increase of *Acidimicrobiales* and *iii1-15*. Interestingly, all of dominant families and genuses generally showed relatively higher abundance in competitive treatments like CB, GB, and GCB. These results suggested that plant competition had a sensitive effect on dominant populations affiliated with different taxa levels, which likely play an important role in driving changes in rhizospheric bacterial structure and improving soil-nutrient condition by rapidly decomposing soil organic matter during ecological restoration on the Loess Plateau.

### Response of Plant Growth and Competition to Rhizospheric Bacterial Community

Relative yield total is an index to quantify the extent to which components of a mixture compete for common limiting resources (De Wit, 1960; Snaydon, 1991). In our study, *RYT* value in three mixture treatments were closed to 2 (1.992, 1.869, 1.942), indicating that two components almost did not compete and show full resource complementarity. The competitive ratio (*CR*) gives the exact degree of competition by indicating the number of times one component is more competitive than the other (Willey and Rao, 1980). *CR_ij_* value is less than 1 means *i* is less competitive than *j*, therefore, the competitive degree of three grasses in this study presented as *S. viridis* < *S. bungeana* < *B. ischaemum*. This result was in concordance with the natural phenomena, as we found, *S. viridis* was earlier successional species, whereas *S. bungeana* and *B. ischaemum* were dominant at the middle and later successional stages, and *B. ischaemum* were C4 plant having larger photosynthetic capacity and environmental adaptability (Zhang, 2005; Wang, 2006; Wang et al., 2009).

Rhizospheric bacterial community imposed an effect on plant growth and competition. As we see, the biomass of *B. ischaemum* in monoculture was less than that in mixture treatments (**Table 2**), and treatments of B, CB, GB, and GCB have apparently higher abundance of family *Rhodospirillaceae*, and treatments of B, GB, and GCB have obviously higher abundance of genus *Rhodobacter* (**Figure 3**). Our results indicated that *B. ischaemum* tended to grow better when lived together with other species, and the unique bacterial communities associated with *B. ischaemum* facilitated the improvement of competitive ability. Genus *Rhodobacter* family *Rhodospirillaceae* (order *Rhodobacterales*, class *Alphaproteobacteria*) are photosynthetic bacterium and have exuberant vitality, which can grow photoheterotrophically under anoxic conditions in the light, chemoheterotrophically in the dark, or heterotrophically under aerobic/microaerobic conditions (Baldani et al., 2014). Due to *Rhodobacter* can encode proteins involved in numerous energy-generating and energy-utilizing processes, such as photosynthesis, carbon fixation, nitrogen fixation, hydrogen utilization, aerobic and anaerobic respiration, denitrification, electron transport and aerotaxis (Madigan et al., 1984; Elsen et al., 2004), the growth and metabolism of these bacterium play an irreplaceable role in carbon and nitrogen cycle as well as material transformation. Therefore, we believe that *B. ischaemum* outcompete other grass species and become the dominant species in the late natural succession stage was attributed to not only the strong competitiveness and adaptiveness but also the function of photosynthetic bacterium cultivated by itself.

### Response of Bacterial Communities to Soil Properties

The CCA indicated that the abundances of the dominant sub-groups of *Proteobacteria, Actinobacteria, Acidobacteria* and *Bacteroidetes* were greatly affected by some soil properties. Results of CCA variance partitioning of environmental factors to the microbial communities indicated that SOC, WSOC, NO_3_^-^-N and AP contents were important driving factors for the formation and transformation of the rhizospheric communities. The SEM model supported the CCA result and indicated that grass competition accounted for 45, 15, 34, and 15% of the variability in SOC, WSOC, NO_3_^-^-N and AP contents, respectively. Furthermore, SOC, WSOC, NO_3_^-^-N, and AP contents together explained 80% of the variability of bacterial structure and 45% of the variability of bacterial richness. Belowground dry weight showed positive effect on SOC, WSOC, NO_3_^-^-N contents and negatively affected AP content. Change of bacterial structure was thus mainly resulted from the direct and positive effects of SOC, WSOC, NO_3_^-^-N, and AP content, and change of bacterial richness was resulted from the positive effect of SOC, WSOC, and AP contents and negative effect of NO_3_^-^-N content.

Most of the *Proteobacteria* sub-groups were positively correlated with factors associated with carbon, and the dominant subgroups of *Actinobacteria* and *Acidobacteria* was opposite, suggesting that most members of this phylum were sensitive to the differences in soil carbon. The presence of grass on bare land will provide organic carbon and nitrogen to decomposer organisms in the form of rhizodeposition and dead biomass, which will subsequently favor copiotrophic groups such as *Proteobacteria* (Goldfarb et al., 2011; Philippot et al., 2013), and defer oligotrophic groups such as *Actinobacteria* and *Acidobacteria* (Fierer et al., 2007, 2012). In addition, *Sphingomonadales, Xanthomonadales*, and *Cytophagales* was positively correlated with NO_3_^-^-N and AP contents. This result suggested that NO_3_^-^-N and AP contents also plays an important role in the formation of rhizospheric bacterial communities, and Zhang et al. (2016) found a close correlation between AP content and bacterial structure. The bacterial structure in our study, however, was poorly correlated with NH_4_^+^-N, TN, and TP contents and pH value. These indices did not differ significantly after the 4-month pot experiment and remained within a narrow range, which may have been responsible for the weak correlations.

## Conclusion

Interactions between plant and soil communities existed in a short-term competitive experiment. Mixture treatments associated with *B. ischaemum* increased the relative abundance of *Proteobacteria* and *Bacteroidetes* and decreased the relative abundance of *Actinobacteria* and *Acidobacteria*. In turn, photosynthetic bacterium, Genus *Rhodobacter* family *Rhodospirillaceae*, affected the growth condition and contributed to increased the competitive ability of *B. ischaemum*. In addition, plant composition caused the difference of SOC, WSOC, NO_3_^-^-N and AP contents, which explained most of the differences in the rhizospheric bacterial communities. Therefore, this interaction between plant composition and soil was in part responsible for plant competitive result and may be the key driving factor to impact and change the trend of vegetation succession on the Loess Plateau of China.

## Author Contributions

CS, SX, and GL initiated and designed the research, collected the materials, and performed the experiments. SX and GL obtained funding for this study. CS analyzed the data and wrote the manuscript. All authors read and approved the manuscript.

## Conflict of Interest Statement

The authors declare that the research was conducted in the absence of any commercial or financial relationships that could be construed as a potential conflict of interest. The reviewer GY declared a shared affiliation, with no collaboration, with the authors to the handling Editor at time of review.

## Footnotes

1 http://drive5.com/uchime/uchime_download.html
